# 4-week eicosapentaenoic acid-rich fish oil supplementation partially protects muscular damage following eccentric contractions

**DOI:** 10.1186/s12970-021-00411-x

**Published:** 2021-03-01

**Authors:** Yosuke Tsuchiya, Hisashi Ueda, Kenichi Yanagimoto, Ayaka Kato, Eisuke Ochi

**Affiliations:** 1 Laboratory of Health and Sports Sciences, Meiji Gakuin University, Kanagawa, Japan; 2 Faculty of Health and Medical Science, Teikyo Heisei University, Chiba, Japan; 3 Food Function R&D Center, Nippon Suisan Kaisha, Ltd, Tokyo, Japan; 4 Faculty of Bioscience and Applied Chemistry, Hosei University, 3-7-2, Kajino, Koganei, 184-8584 Tokyo, Japan; 5 Graduate School of Sports and Health Studies, Hosei University, Tokyo, Japan

**Keywords:** Eicosapentaenoic acid, Omega-3, Sports nutrition, Ergogenic aid, Long‐chain n-3 polyunsaturated fatty acids, Lengthening, Muscle function, Joint flexibility

## Abstract

**Background:**

We previously showed 8-week of fish oil supplementation attenuated muscle damage. However, the effect of a shorter period of fish oil supplementation is unclear. The present study investigated the effect of fish oil, eicosapentaenoic acid (EPA) and docosahexaenoic acid (DHA), for 4 weeks on muscular damage caused by eccentric contractions (ECCs) of the elbow flexors.

**Methods:**

Twenty-two untrained men were recruited in this double-blind, placebo-controlled, parallel design study and the subjects were randomly assigned to the EPA and DHA group (EPA and DHA, *n* = 11) and placebo group (PL, *n* = 11). They consumed either EPA 600 mg and DHA 260 mg per day or placebo supplement for 4 weeks prior to exercise. Subjects performed 60 ECCs at 100 % maximal voluntary contraction (MVC) using a dumbbell. Changes in MVC torque, range of motion (ROM), upper arm circumference, muscle soreness, echo intensity, muscle thickness, serum creatine kinase (CK), and interleukin-6 (IL-6) were assessed before exercise; immediately after exercise; and 1, 2, 3, and 5 days after exercise.

**Results:**

ROM was significantly higher in the EPA and DHA group than in the PL group immediately after performing ECCs (*p* < 0.05). No differences between groups were observed in terms of MVC torque, upper arm circumference, muscle soreness, echo intensity, and thickness. A significant difference was observed in serum CK 3 days after ECCs (*p* < 0.05).

**Conclusions:**

We concluded that shorter period EPA and DHA supplementation benefits joint flexibility and protection of muscle fiber following ECCs.

## Introduction

Omega-3 polyunsaturated fatty acids include of eicosapentaenoic acid (EPA; 20:5 n-3) and docosahexaenoic acid (DHA; 22:6 n-3), which are mainly contained in fish oil. EPA and DHA are known to have anti-inflammatory effects and increased red blood cell (RBC) deformability as a consequence of incorporation of omega-3 polyunsaturated fatty acid into RBC membrane phospholipids [1]. Recently, omega-3 polyunsaturated fatty acid supplementation has been proposed as an ergogenic aid for athletes [2].

Exhaustive eccentric contractions (ECCs) or unaccustomed exercise causes delayed onset muscle soreness (DOMS), reduction in maximal strength, limitation of range of motion (ROM), and muscle swelling [3]. In addition, previous studies showed that after ECCs, serum myoglobin (Mb), creatine kinase (CK), and interleukin (IL)-6 increase [3–7]. ECC-induced muscle damage is defined as morphological changes in the sarcomeres and endomysium and inflammatory responses in muscle fibers and connective tissues [8]. Previous studies have reported that EPA and DHA supplementation positively affect these symptoms of muscle damage [7, 9–12]. Consumed omega-3 polyunsaturated fatty acids are incorporated into phospholipid, a major component of the cell membrane, and have been reported to inhibit the effects of inflammation and reactive oxygen species [13]. Helge et al. [14] reported that the total proportion of omega-3 polyunsaturated fatty acids in muscle cellular membrane was significantly increased after 4 weeks of high-fat diet in rat. It is assumed that ingestion of EPA and DHA alleviates exercise-induced muscle damage by the incorporation of omega-3 polyunsaturated fatty acids into the muscle cell membrane.

We previously examined 30 ECCs in elbow flexors after an intake of both 600 mg/day of EPA and 260 mg/day of DHA for 8 weeks [7] and showed the inhibitions in reduced muscle strength and ROM, and increased IL-6 as inflammatory response [7]. Furthermore, other studies have reported that ingestion of 8-week EPA (600 mg/day) and DHA (260 mg/day) attenuates nerve dysfunction [9] and muscle stiffness [11] following 60 ECCs using a dumbbell at 100 % 1RM. However, the study that conducted these experiments in shorter periods (< 8 weeks) is controversial and insufficient [10, 12, 15]. Specifically, Tartibian et al. [10, 12] examined the effects of 324 mg/day EPA and 216 mg/day DHA ingestion for 30 days on muscle damage after 40 min of bench stepping. As the results, development of DOMS, limited ROM, muscle swelling, and elevated of serum CK and IL-6 were inhibited by the ingestions. On the other hand, it showed that the ingestion of 400 mg/day EPA and 270 mg/day DHA for 30 days were not effective to DOMS, ROM, and serum CK and IL-6 following 50 maximal ECCs of elbow flexors [15]. Hence, elucidating the efficacy of shorter period of EPA and DHA supplementation is important for athletes and resistance training enthusiasts.

Therefore, the present study examined whether 600 mg/day of EPA and 260 mg/day of DHA supplementation for 4 weeks reduce muscle damage following elbow flexors in ECCs. We hypothesized that short term EPA and DHA supplementation may improve ECC-induced muscle damage.

## Methods

### Subjects

A total of 22 healthy recreational untrained men were recruited for this study. The participants were not allergic to fish and had not participated in any regular resistance training experience for at least one year before this study. Further, participants were asked not to participate in other clinical trials and interventions, such as massage, stretching, strenuous exercise, excessive consumption of food or alcohol, and intake of supplementations or medications during the experimental period. All participants were provided with detailed explanations of the study protocol prior to participation, and informed consent was obtained from all participants. The present study was performed in accordance with the Declaration of Helsinki and was approved by the ethics committee for human experiments of Teikyo Heisei University (ID: R01-040). Moreover, the study was registered at the University Hospital Medical Information Network Clinical Trials Registry (UMIN-CTR identifier: UMIN000038003).

### Study design

The study used the double-blind, placebo-controlled, parallel-group trial design. The participants were randomly assigned to two groups using a table of random numbers to minimize the intergroup differences in terms of age, body fat, and body mass index (BMI). The placebo (PL) and EPA and DHA group consumed daily placebo or fish oil capsules for 4 weeks prior to an exercise experiment and for 5 days after the exercise experiment. The sequence allocation concealment and blinding of participants and researchers were maintained throughout this period. Medication adherence was assessed using the daily record of the patients and via pill count at the end of the study. On the day of exercise testing, muscle damage markers were assessed using the nondominant arm before exercise. Immediately after these baseline measurements, the participants performed ECCs using the same arm. Maximum voluntary contraction (MVC) torque, ROM, DOMS, circumference, muscle echo intensity, and thickness were measured immediately before and after exercise and 1, 2, 3, and 5 days after exercise. Serum CK and IL-6 were measured before exercise and 1, 2, 3, and 5 days after exercise. In addition, we measured serum fatty acid levels at before and after 4 weeks supplementation. Subjects were instructed to eat a light meal > 2 h before arriving at the laboratory. In addition, they were asked to refrain from any exercise for 24 h, before the study visit. In addition, we assessed the nutrition status of all participants prior to supplement consumption and after the experimental testing on food frequency using a questionnaire based on food groups (FFQg version 3.5, Kenpakusha, Tokyo, Japan). Furthermore, we measured serum fatty acid levels, including EPA, DHA, arachidonic acid (AA), and dihomo-gamma-linolenic acid (DGLA) levels.

### Supplements

The EPA and DHA group consumed eight 300-mg EPA-rich fish oil softgel capsules (Nippon Suisan Kaisha Ltd., Tokyo, Japan) per day, and the total consumption was 2,400 mg per day (600-mg EPA and 260-mg DHA). The PL group consumed eight 300-mg corn oil softgel capsules per day (without EPA and DHA), and the total consumption was 2,400 mg. The participants consumed the capsules within 30 min after the morning meal.

### Blood sample

The participants fasted for 8 h before a trained doctor obtained blood samples from their forearms. The blood samples were allowed to clot at room temperature (25 °C) and were then centrifuged at 3,000 rpm for 10 min at 4 °C. The serum was extracted and stored at − 20 °C until analysis. The serum levels of DGLA, AA, EPA, and DHA were measured. In addition, we evaluated serum creatine kinase (CK) and interleukin-6 (IL-6) as muscle damage markers, as previously described [7].

### Eccentric contractions

For the ECCs, the participant sat on a preacher curl bench with his shoulder joint angle at 45° flexion. For the use of the dumbbell, the value of maximal voluntary contraction (MVC) torque measurement at 90° was converted to kilograms. The exercise comprised six sets of 10 maximal voluntary ECCs of the elbow flexors with a rest period of 90 s between each set, as described in our previous study [11]. The dumbbell was handed to the participant at the elbow flexed position (90°), and the participant was instructed to lower it to a fully extended position (0°) at an approximately constant speed (30°/s) in time (3 s) with a metronome. The investigator then removed the dumbbell, and the participant returned his arm without the dumbbell to the start the position for the next ECCs.

### Maximum voluntary contraction torque

For the measurement of MVC torque, each subject was seated with nondominant arm attached to a custom-designed ergometer and that performed isometric of the elbow flexors. The MVC torque was measured three 3-s MVCs at 90° and 110° of elbow joint angle with a 30-s rest during contractions. Subjects were verbally encouraged to give their maximal effort during the muscle strength tests. The greatest 1-s average of the three trials for each angle was used for subsequent analysis. The peak torque of each angle contraction was used as the MVC torque. The torque signal was amplified using a strain amplifier (LUR-A-100NSA1; Kyowa Electronic Instruments, Tokyo, Japan). The analog torque signal was converted to digital signals using a 16-bit analog-to-digital converter (Power-Lab 16SP; AD Instruments, Bella Vista, Australia). The sampling frequency was set at 10 kHz. The test–retest reliability of the MVC measures based on coefficient of variation (CV) was 2.8 %.

### Range of motion of the elbow joint

To examine the ROM of the elbow joint, two elbow joint angles (extended and flexed) were measured using a goniometer (Takase Medical, Tokyo, Japan). The extended joint angle was recorded while the participant attempted to fully extend the joint with the elbow held by his side and the hand in supination [7, 16, 17]. The flexed joint angle was identified while the participant attempted to fully flex the joint from an equally fully extended position with the hand supinated. The ROM was calculated by subtracting the flexed joint angle from the extended joint angle. The test–retest reliability of the ROM measures based on CV was 2.2 %.

### Muscle soreness

Muscle soreness in the elbow flexors was assessed using a 10-cm visual analogue scale in which 0 indicated “no pain” and 10 indicated “unbearable imaginable” [7, 16, 17]. The participant relaxed his arm in a natural position. The investigator then palpated the upper arm using a thumb, and the participant indicated his pain level using the visual analogue scale. All tests were conducted by the same investigator who had been trained to use the same pressure over time between participants. The test–retest reliability of the VAS measures based on CV was 1.9 %.

### Upper arm circumference

Upper arm circumference was assessed at 9 cm above the elbow joint using a tape measure while the participants were standing with their arms relaxed by their side [9]. The measurement marks were maintained during the experimental period using a semipermanent ink marker, and a well-trained investigator obtained the measurements. The average value of the three measurements was used for further analysis. The test-retest reliability of the circumference measurements based on CV was 2.2 %.

### Muscle echo intensity and thickness

B-mode ultrasound pictures of the upper arm were obtained using the biceps brachii via an ultrasound (SONIMAGE HS1, Konika Minolta, Japan), and the probe was placed 9 cm from the elbow joint at the position marked for the measurement of the upper arm circumference. The same gains and contrast were used over the experimental period. The transverse images were transferred to a computer as bitmap (.bmp) files and analyzed using a computer. The average muscle echo intensity of the region of interest (20 × 20 mm) was calculated using the computer image analysis software that provided a grayscale histogram (0, black; 100, white) for the region, as described in a previous study (ImageJ, Maryland, USA) [9]. Scanned images of each muscle were transferred to a personal computer and the thickness of biceps rachii was manually calculated via tracing using same software. The test-retest reliability of the muscle echo intensity and thickness measurements based on CV were 2.1 % and 1.4 %, respectively.

### Statistical analyses

All analyses were performed using the SPSS software version 25.0 (IBM Corp., Armonk, NY). Values were expressed as means ± standard deviation. MVC torque, ROM, circumference, echo intensity, and thickness of values before exercise to 5 days post-exercise were calculated based on relative changes from the baseline. MVC, ROM, visual analogue scale (VAS), circumference, echo intensity, thickness, and blood data of the PL and EPA and DHA groups were compared using two-way repeated-measure analysis of variance (ANOVA). When a significant primary effect or interaction was found, Bonferroni’s correction was performed for post-hoc testing. A p-value of < 0.05 was considered statistically significant.

## Results

No significant differences were observed between the EPA and DHA and PL groups in terms of age, weight, and BMI (PL group, n = 11; age, 19.8 ± 1.5 years; height, 169.0 ± 7.8 cm; weight, 65.4 ± 8.4 kg; body fat, 15.7 ± 7.6 %; and BMI, 23.2 ± 3.3 kg/m^2^; EPA and DHA group, n = 11; age, 20.2 ± 0.4 years; height, 167.4 ± 5.4 cm; weight, 65.0 ± 8.9 kg; body fat, 17.2 ± 6.9 %; and BMI, 23.2 ± 2.9 kg/m^2^). Based on the results of the food frequency survey, no difference was observed in the PL group between before (energy, 1953.6 ± 442.2 kcal; protein, 64.4 ± 16.9 g; fat, 64.6 ± 12.3 g; carbohydrate, 263.5 ± 72.5 g; and omega-3 fatty acid, 1.8 ± 0.2 g) and after supplementation (energy, 1991.0 ± 556.2 kcal; protein, 64.9 ± 22.2 g; fat, 68.3 ± 24.1 g; carbohydrate, 262.0 ± 77.4 g; and omega-3 fatty acid, 2.0 ± 0.7 g) and in the EPA and DHA group between before (energy, 1907.9 ± 349.7 kcal; protein, 70.9 ± 14.3 g; fat, 68.1 ± 12.7 g; carbohydrate, 243.8 ± 73.4 g; and omega-3 fatty acid, 2.1 ± 0.6 g) and after supplementation (energy, 1894.9 ± 431.4 kcal; protein, 70.7 ± 17.3 g; fat, 65.8 ± 13.7 g; carbohydrate, 244.1 ± 81.9 g; and omega-3 fatty acid, 2.0 ± 0.6 g). These data reveal that physical characteristics and nutrition status of the participants did not change during the experimental period.

### Polyunsaturated fatty acids

As shown in Table 1, no significant changes were observed in the PL group before and after supplementation in terms of DGLA, AA, EPA, and DHA levels. In the EPA and DHA group, blood EPA level increased after 4 weeks (*p* < 0.05). However, no significant difference was observed in the DGLA, AA, and DHA levels. For comparison between groups, the EPA level was significantly higher in the EPA and DHA group than in the PL group after the 4-week supplementation (*p* < 0.05).

**Table 1:** Changes of serum dihomo-gamma-linolenic acid, arachidonic acid, eicosapentaenoic acid, and docosahexaenoic acid at before and after 4-week

		Dihomo-gamma-linolenic acid (µg/ml)	Arachidonic acid (µg/ml)	Eicosapentaenoic acid (µg/ml)	Docosahexaenoic acid (µg/ml)
Placebo	Before	49.0 ± 9.9	189.1 ± 40.6	20.9 ± 9.2	68.5 ± 24.6
	After	42.9 ± 1.8	198.7 ± 39.5	14.2 ± 6.1	59.4 ± 22.3
EPA	Before	44.7 ± 9.3	201.6 ± 38.1	33.5 ± 21.2	70.9 ± 18.0
	After	42.3 ± 10.5	186.8 ± 20.3	58.2 ± 25.7 *†	78.2 ± 17.7

* *p* < 0.05, Compare with placebo group for after supplementation, †*p* < 0.05, Compare with EPA and DHA group for before supplementation

### Maximal voluntary isometric contraction torque

**Fig. 1:**
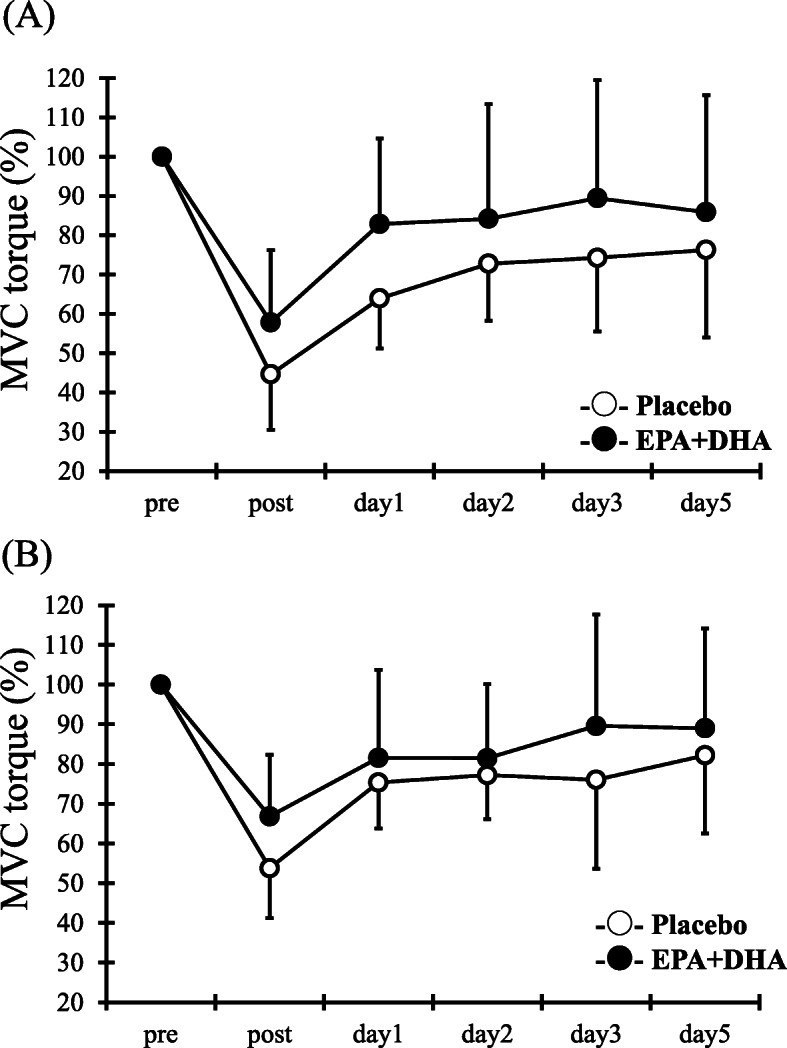
Changes (mean ± SD) of maximal voluntary isometric contraction (MVC) torque at 90° (**a**), and 110° (**b**) measured before (pre) and immediately after (post) the eccentric contractions exercise and 1, 2, 3 and 5 days in the placebo (PL) and EPA and DHA groups

For the MVC, although a times main effect was observed (90°: *F* = 28.8, p < 0.05, 110°: *F* = 20.8, *p* < 0.05), but not a main effect for group (90°: *F* = 2.5, 110°: *F* = 1.4) and interaction effect for times and group (90°: *F* = 1.2, 110°: *F* = 0.9) (Fig. 1).

### Range of motion of the elbow joint

**Fig. 2:**
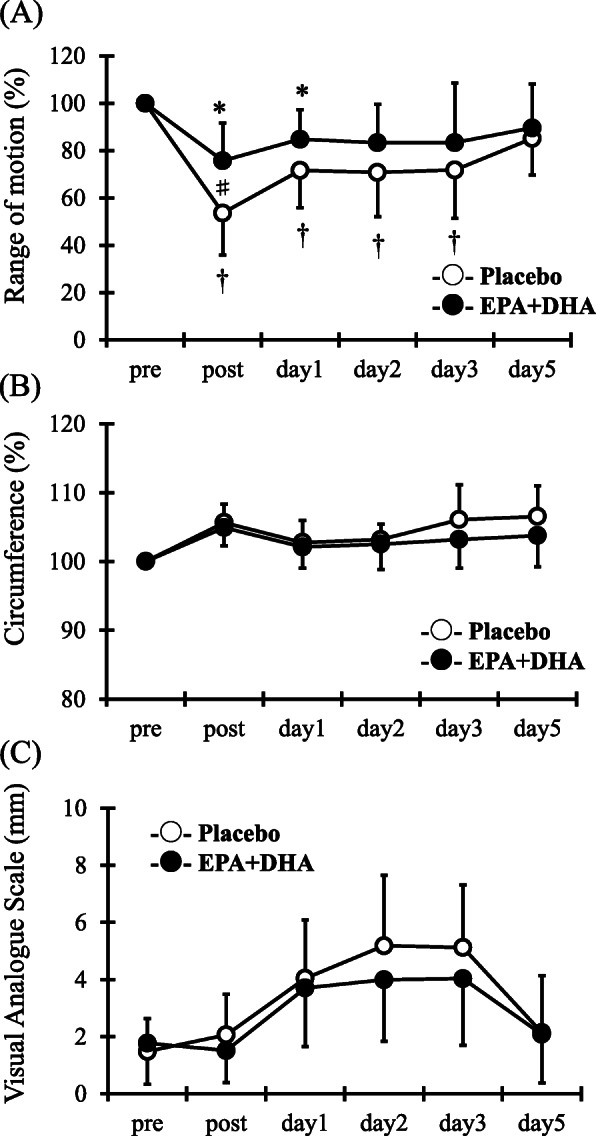
Changes (mean ± SD) of range of motion (ROM) (**a**), circumference (**b**), and visual analogue scale (VAS) (**c**) measured before (pre) and immediately after (post) the eccentric contractions exercise and 1, 2, 3 and 5 days in the PL and EPA and DHA groups. # *p* < 0.05 for the difference between groups; † *p* < 0.05 for the difference from the pre-exercise value in the PL group, * *p* < 0.05 for the difference from pre-exercise value in the EPA and DHA group

As shown in Fig. 2a, ANOVA revealed an interaction (*F* = 3.4, *p* < 0.05), times (*F* = 20.6, *p* < 0.05), and group effect (*F* = 4.0, *p* < 0.05) for ROM. Post-hoc analysis revealed that a significant decrease in ROM was observed in the PL group immediately after exercise, which remained lower than the baseline on days 1, 2, and 3 after exercise. The ROM in the EPA and DHA group decreased immediately after and 1 day after exercise compared with the pre-exercise value but returned to baseline on day 2 after exercise. The ROM in the EPA and DHA group was significantly higher than that of the PL group immediately after exercise (EPA and DHA group; 76.5 ± 16.7 %, PL group; 53.1 ± 18.7 %; *p* < 0.05).

### Upper arm circumference and muscle soreness

For upper arm circumference and muscle soreness, although a times main effect was observed (circumference: *F* = 11.8, p < 0.05, muscle soreness: *F* = 17.7, p < 0.05), but not a main effect for group (circumference: *F* = 3.4, muscle soreness: *F* = 0.7) and interaction effect for times and group (circumference: *F* = 1.7, muscle soreness: *F* = 1.0) (Fig. 2b and c).

### Muscle thickness and echo intensity

**Fig. 3:**
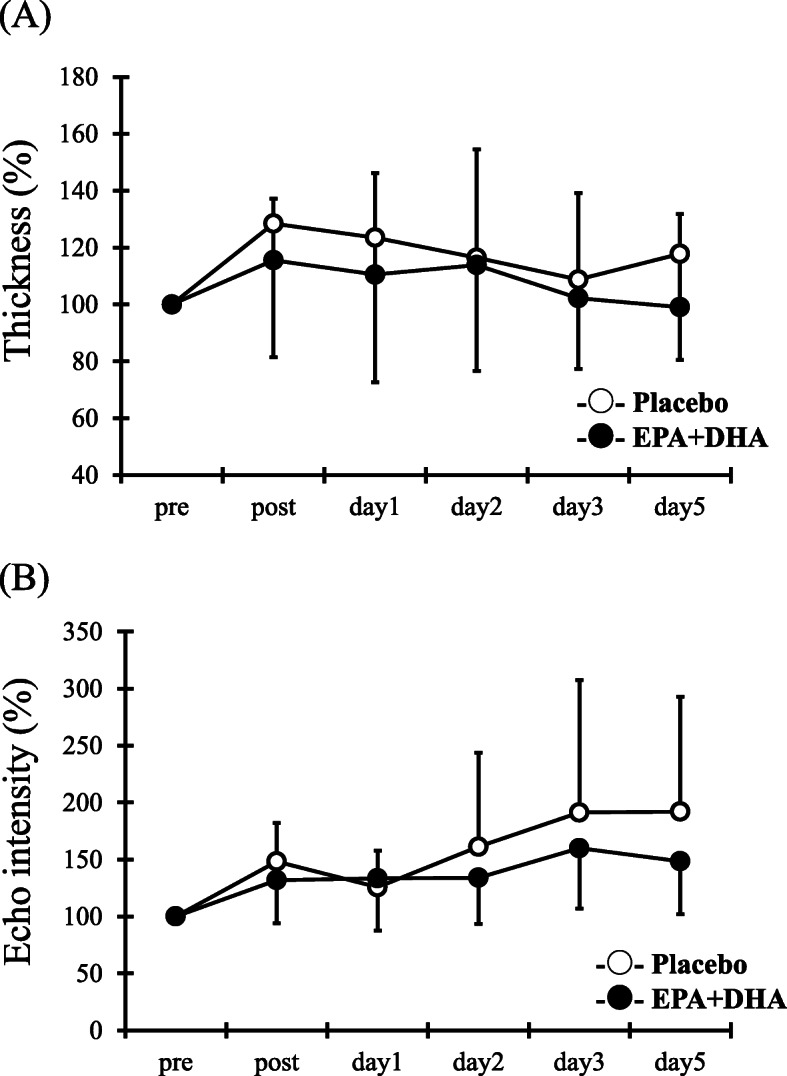
Changes (mean ± SD) of muscle thickness (**a**) and muscle echo intensity (**b**) measured before (pre) and immediately after (post) the eccentric contractions exercise and 1, 2, 3 and 5 days in the PL and EPA and DHA groups

For muscle thickness and echo intensity, although a times main effect was observed (muscle thickness: *F* = 3.1, *p* < 0.05, echo intensity: *F* = 7.5, *p* < 0.05), but not a main effect for group (muscle thickness: *F* = 0.2, echo intensity: *F* = 1.7) and interaction effect for times and group (muscle thickness: *F* = 0.5, echo intensity: *F* = 1.2) (Fig. 3a and b).

### Blood serum analysis

**Fig. 4:**
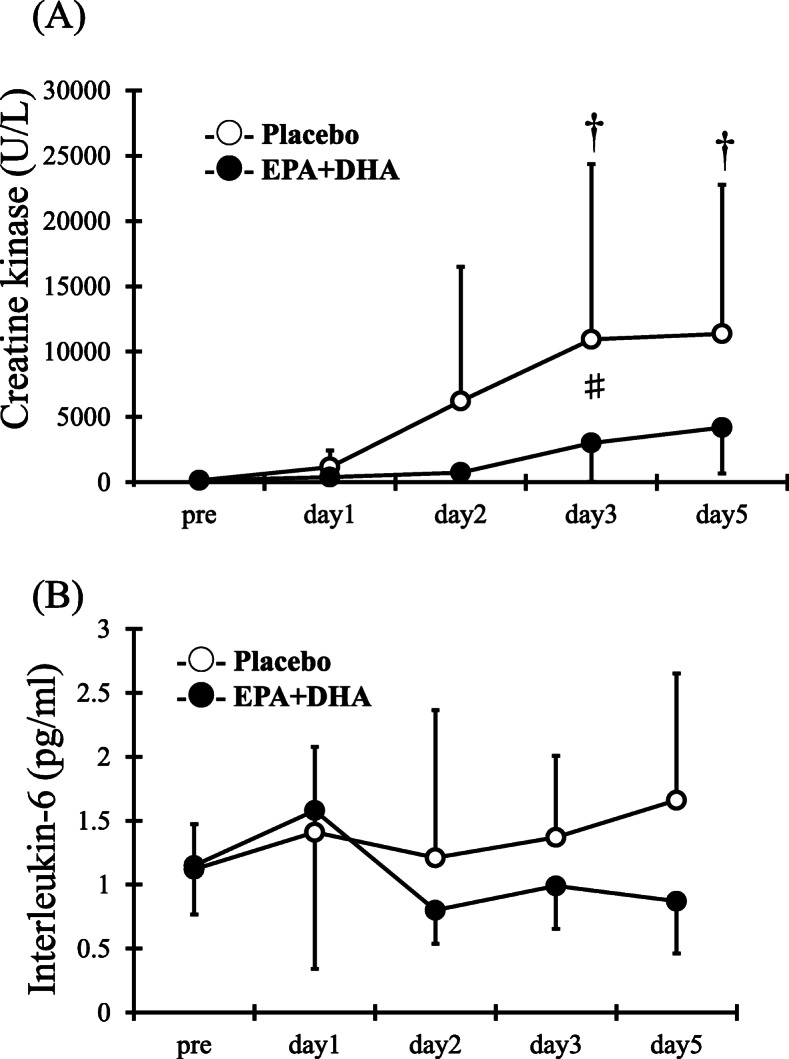
Changes (mean ± SD) of serum creatine kinase (**a**) and serum interleukin-6 (**b**) measured before (pre) and the eccentric contractions exercise and 1, 2, 3 and 5 days in the placebo (PL) and EPA and DHA groups. # *p* < 0.05 for the difference between groups; † *p* < 0.05 for the difference from the pre-exercise value in the PL group

ANOVA revealed an interaction (*F* = 3.1, *p* < 0.05), times (*F* = 8.0, *p* < 0.05), and group effect (*F* = 4.7, *p* < 0.05) for serum CK. Serum CK levels in the PL group significantly increased 3 and 5 days after exercise compared with pre-exercise values (Fig. 4a, *p* < 0.05). However, no significant difference after exercise compared with pre-exercise values was observed in the EPA and DHA group. Serum CK levels were significantly higher in the PL group than the EPA and DHA group 3 days after exercise (PL, 12132.7 ± 13652.2 U/L vs. EPA, 2575.2 ± 2798.9 U/L, p < 0.05). In contrast, no interaction (*F* = 2.2), times (*F* = 17.5), and group effect (*F* = 2.3) in IL-6 were detected between times and the group (Fig. 4b).

## Discussion

This study investigated the effect of consuming EPA-enriched fish oil for 4 weeks on temporal muscular dysfunction and soreness following ECCs. As a result, the 4-week ingestion of 600-mg EPA and 260-mg DHA attenuated the loss of joint flexibility after 60 ECCs in the elbow flexors. Moreover, we confirmed the inhibition of increased blood CK level, suggesting protection of muscle damage to muscle fibers. However, no effects were observed on loss of muscle strength, delayed onset muscle soreness, muscle swelling, echo intensity, and blood IL-6. These results suggest that supplementation with EPA-enriched fish oil for 4 weeks is effective to a limited extent for attenuating acute exercise-induced muscular damage. These results support our hypothesis.

Herein, we confirmed that EPA (600 mg/day) and DHA (260 mg/day) consumed for 4 weeks significantly attenuated reduced ROM after 60 ECCs in the elbow flexors. The decreased ROM after ECCs has been attributed to the increased passive muscle stiffness due to inflammatory response in myofibrils, elevated cytoplasmic calcium levels, and muscle swelling due to inflammatory reactions [18, 19]. In our previous study, 30 isokinetic ECCs produced by elbow flexion with maximum effort were loaded after 600-mg EPA and 260-mg DHA per day for 8 weeks before exercise [7]. As a result, the reduction of ROM in the elbow joint until 3 days after exercise in EPA and DHA group was significantly less compared with that in the placebo group [7]. In a different study, the 8-week consumption of 600 mg/day EPA and 260 mg/day DHA significantly reduced elbow ROM until 5 days after 60 ECCs using dumbbells at 100 % MVC [11]. As a study investigating the effectiveness of supplementation for a period shorter than 8 weeks, Lenn et al. [15] tested the effect of 30-day consumption of 1,800-mg/day omega-3 fatty acids (398-mg EPA and 269-mg DHA) on muscle damage following 50 ECCs by elbow flexions in 21 untrained men and women. The results of that study show no differences in the loss of ROM in the elbow between the omega-3 fatty acid group and the placebo group. As consumption of 2,400-mg/day omega-3 fatty acids (600-mg EPA and 260-mg DHA) was effective in this study, the dose, rather than the duration of administration, appears to be a more important factor determining the preventive effect on joint flexibility. In a review article [20], it has been suggested that an ingestion ratio of EPA to DHA of approximately 2:1 may be beneficial in counteracting exercise-induced inflammation, which is almost similar to our ratio. Therefore, ratio of EPA to DHA may be also important factor to maintain the joint flexibility after muscle damage. In addition, a study involving 24 healthy young men has reported that reduced ROM in the knee joint after loading ECCs of quadriceps through a 40-min bench stepping exercise was significantly reduced in those who consumed 324-mg/day EPA and 216-mg/day DHA for 30 days [10]. In that study, ingestion of lower EPA and DHA was effective presumably because the multi-joint movement in lower limbs for a long period of time was a relatively low-intensity exercise. These data suggest that the effectiveness of short-term supplementation with EPA and DHA (28–30 days) on joint flexibility may differ depending on the omega-3 fatty acid dose, mode of exercise, and specific muscles involved.

In the present study, no differences in IL-6 between the groups were observed, and blood CK elevation in the EPA and DHA group was significantly reduced. The increase in CK following ECCs is attributable to micro-damage to muscle fibers [3, 5]. A study involving mice has reported that the amount of omega-3 fatty acids in the cell membrane significantly increased after 3-week administration of omega-3 fatty acids [21]. Therefore, a possible reason why the exercise-induced CK elevation was mitigated herein is that omega-3 fatty acids were incorporated into and protected cell membrane in muscle fiber. Similarly, Tartibian et al. [10] have reported that consumption of EPA and DHA for 30 days reduced CK elevation after ECCs from 40 min of the bench stepping exercise. Furthermore, some studies have demonstrated that consumption of EPA and DHA reduced the elevation of IL-6, an inflammatory cytokine [7, 12, 22, 23]. Possible reasons why our results did not agree with these previous studies were used different modes such as isokinetic ECCs [7, 22], arm carl machine [23], or bench stepping exercise [12]. In addition, Phillips et al. [23] showed that there were significant differences between supplementation of DHA group and placebo for IL-6 at 10 days after exercise. Future studies considering exercise modes and measurement of time point are necessary.

The present study also indicated no effect of 4-week supplementation with EPA and DHA on DOMS. A study has reported that the results of daily ingestion of 2,000-mg EPA and 1,000-mg DHA for 2 weeks significantly mitigated DOMS in the upper arm after two sets of ECCs (until exhaustion) via elbow flexions using dumbbells at an intensity of 120 % 1RM [24]. Similarly, as a result of 3,600 mg of EPA and DHA ingested for 2 weeks have also been reported to mitigate DOMS in the upper arm and thigh after 10 sets of ECCs (until exhaustion) through elbow flexions and knee extensions at an intensity of 50 % 1RM [25]. Studies using 8-week consumption of EPA and DHA at lower doses (600 mg/day of EPA and 260 mg/day of DHA) have reported significant mitigation of DOMS after 30 isokinetic ECCs by elbow flexions [7] and DOMS after isotonic ECCs using dumbbells at an intensity of 40 % 1RM [9]. In contrast, 30-day consumption of 287-mg/day EPA and 194-mg/day DHA [15] and 6-week consumption of 1,300-mg/day EPA and 300-mg/day DHA [26] have been demonstrated to be ineffective for DOMS following isokinetic ECCs. Therefore, we suggest that ingestion at a high daily dose or for a long period of time appears to be important for EPA and DHA to exert the mitigation effect on DOMS.

Herein, loss of muscle strength, muscle swelling, and increased echo intensity after exercise were not prevented by the 4-week ingestion of EPA and DHA. In a previous study, we have reported that the results of 8-week ingestion at similar doses to those used in the present study reduced the loss of muscle strength [7, 9, 11]. Furthermore, 30-day ingestion of 287-mg/day EPA and 194-mg/day DHA [15] and 6-week ingestion of 1,300-mg EPA and 300-mg DHA [26] have been shown to be ineffective against loss of muscle strength. Considering the results of our present and previous studies, the duration of EPA and DHA intake is an important determinant of the effectiveness for muscle strength after exercise. One of reasons for the importance of duration, previous study showed that the increase of omega-3 polyunsaturated fatty acids in muscle cellular membrane needs over 4 weeks in rat [14]. Therefore, we speculate there is no effect to muscle strength, showing the different and multiple roles of EPA and DHA for muscle damage. In addition, no consensus has been obtained thus far on the swelling of muscles. Consumption of EPA (600 mg/day) and DHA (260 mg/day) for 8 weeks has been reported to attenuate muscle swelling after ECCs through elbow flexions at an intensity of 100 % MVC [11]. Ingestion of 324-mg EPA and 216-mg DHA per day for 30 days has been reported to mitigate muscle swelling after ECCs in lower limbs [10]. Several previous studies show that consumption of EPA and DHA for 2–8 weeks did not attenuate muscle swelling after ECCs [7, 9, 24, 25]. In many studies, muscle swelling was not evaluated directly and was measured using a measuring tape. The use of MRI or ultrasonography may be necessary for evaluation in the future. In the ultrasound measurement, an increased echo intensity reflects the amount of free water or edema due to disintegration of the extracellular matrix [6]. Our previous study has shown that consumption of EPA and DHA for 8 weeks resulted in a smaller increase in echo intensity following elbow flexions [11]. Therefore, more studies are needed to clarify the effect on echo intensity after ECCs because pf the paucity of data; regardless, the available data suggest the importance of the duration of consumption.

## Conclusions

Herein, we confirmed that 4-week 2,400 mg/day fish oil of supplementation with EPA and DHA at daily doses of 600 mg of 260 mg, respectively, alleviated the loss of joint flexibility and the increase in CK after ECCs through elbow flexions. However, other effects for the parameters of ECC-induced muscle damage were limited compared with that of 8-week consumption of EPA and DHA [7, 11]. Therefore, we conclude that supplementation with EPA-enriched fish oil for 4 weeks is effective to a limited extent for attenuating acute exercise-induced muscular damage. Our finding that there is certain duration of supplement to obtain the earlier muscle recovery will be important information to athletes and training enthusiasts acquire efficient training.

## Data Availability

Please contact author for data requests.
